# Comprehensive analyses of a tumor-infiltrating lymphocytes-related gene signature regarding the prognosis and immunologic features for immunotherapy in bladder cancer on the basis of WGCNA

**DOI:** 10.3389/fimmu.2022.973974

**Published:** 2022-09-20

**Authors:** Zexi He, Jun Gu, Ting Luan, Haihao Li, Charles Li, Zhenjie Chen, Enxiu Luo, Jiansong Wang, Yinglong Huang, Mingxia Ding

**Affiliations:** ^1^ Department of Urology, The Second Affiliated Hospital of Kunming Medical University, Kunming, China; ^2^ Urological Disease Clinical Medical Center of Yunnan Province, The Second Affiliated Hospital of Kunming Medical University, Kunming, China; ^3^ Zhongke Jianlan Medical Research Institute, Beijing, China

**Keywords:** tumor-infiltrating lymphocytes, bladder cancer, prognosis, immunity, tumor microenvironment

## Abstract

Tumor-infiltrating lymphocyte (TIL) is a class of cells with important immune functions and plays a crucial role in bladder cancer (BCa). Several studies have shown the clinical significance of TIL in predicting the prognosis and immunotherapy efficacy. TIL-related gene module was screened utilizing weighted gene coexpression network analysis. We screened eight TIL-related genes utilizing univariate Cox regression analysis, least absolute shrinkage and selection operator (LASSO) Cox regression analysis, and multivariate Cox regression analysis. Then, we established a TIL-related signature model containing the eight selected genes and subsequently classified all patients into two groups, that is, the high-risk as well as low-risk groups. Gene mutation status, prognosis, immune cell infiltration, immune subtypes, TME, clinical features, and immunotherapy response were assessed among different risk subgroups. The results affirmed that the TIL-related signature model was a reliable predictor of overall survival (OS) for BCa and was determined as an independent risk factor for BCa patients in two cohorts. Moreover, the risk score was substantially linked to age, tumor staging, TNM stage, and pathological grade. And there were different mutational profiles, biological pathways, immune scores, stromal scores, and immune cell infiltration in the tumor microenvironment (TME) between the two risk groups. In particular, immune checkpoint genes’ expression was remarkably different between the two risk groups, with patients belonging to the low-risk group responding better to immune checkpoint inhibition (ICI) therapy. In conclusion, our study demonstrates that the TIL-related model was a reliable signature in anticipating prognosis, immune status, and immunotherapy response, which can help in screening patients who respond to immunotherapy.

## Introduction

Bladder cancer (BCa) is among the prevalent tumors that affect the urinary system. Due to its high incidence as well as poor prognosis, it is considered the 6^th^ most prevalent malignancy and the ninth primary cause of malignancy-associated mortalities in men globally (1). The standard treatment for patients who have early non-muscular-invasive bladder cancer includes transurethral bladder tumor resection and postoperative bladder perfusion chemotherapy (2), but many patients are still at risk of tumor recurrence and metastasis (3). In addition, although bladder cancer is early detected by some DNA methylation-based urine assays (4), about 30% of patients are initially diagnosed with muscle-invasive bladder cancer, which progresses rapidly and has a poor prognosis (5). Following the advancement and innovation of the latest medical technologies, huge progress has been realized in the management of BCa. At present, there is accumulating evidence that Immune-based therapy, particularly immune checkpoint inhibitors (ICI), as a new treatment method, can benefit BCa patients (6, 7). Consequently, it is extremely important in clinical practice to validate the tumor progression-related molecular mechanism in order to develop new targeted therapeutics to enhance the BCa patients’ prognoses.

Tumor microenvironment (TME) denotes the complex internal environment generated and existed by tumor cells, including cell components, such as tumor cells, fibroblasts, immune cells, and extracellular components such as cytokines, growth factors, and extracellular matrix. There is an interactive relationship between tumors and TME. A tumor can influence its microenvironment by producing cell signaling molecules to enhance tumor angiogenesis and trigger immune tolerance, whereas immune cells in the microenvironment can influence the growth as well as the development of cancerous cells. Tumor-infiltrating lymphocyte (TIL) is a special type of lymphocyte in tumor tissues, including T cells, B cells, NK cells, macrophages, and so on. TIL can affect TME through the interaction between different cells, thus influencing the occurrence and development of a tumor and exerting an anti-tumor effect directly or indirectly (8, 9). In addition, TIL establishes a complex network of intercellular interactions that help maintain the immunosuppressive microenvironment, promoting immune escape and triggering tumors. Tumor-infiltrating lymphocytes are associated with cancer immune regulation and TME, and play a fundamental role in tumor genesis, development, metastasis, and prognosis (10, 11). Therefore, the identification and characterization of TIL-related genes (TILRGs) are crucial for understanding BCa immune cell infiltration, TME, and treatment and prognosis of BCa. In this present investigation, weighted gene coexpression network analysis (WGCNA) and ssGSEA algorithm were utilized to identify TIL and immune checkpoint-related co-expressed genes. Finally, 8 hub genes were identified to create the immunoprognostic model. Then, gene mutation status, clinical features, prognosis, immune cell infiltration, immune subtypes, TME, and immunotherapy response were assessed among different risk subgroups. The results of this study will help in the exploration of the mechanism of TIL immune infiltration in BCa, monitor the immunotherapy response of BCa patients, search for new immunotherapy targets, and enhance the BCa patients’ prognoses.

## Materials and methods

### Data acquisition and processing

TCGA (http://cancergenome.nih.gov/) provided BCa transcriptome sequencing data (transcripts per kilobase (TPM) values), Somatic mutation data, and Clinical information comprising 414 cancerous and 19 normal tissue samples, respectively, through TCGAbiolinks (v2.24.1) (12). Additionally, Copy Number Variation (CNV) data, on the other hand, were provided by the UCSC database (http://xena.ucsc.edu/) and pre-processed utilizing the Perl program (v5.32.1). Genomic, transcriptomic, and clinical information from patients with metastatic urothelial cancer treated with an anti-PD-L1 agent (atezolizumab) were obtained under the Creative Commons 3.0 license and can be downloaded from http://research-pub.gene.com/IMvigor210CoreBiologies (13). The GSE13507 dataset was acquired from the Gene Expression Omnibus (GEO, http://www.ncbi.nlm.nih.gov/geo/) database whose platform is GPL6102 (14). Then, the data were further log2-transformed for subsequent analyses. The baseline clinical characteristics of TCGA, IMvigor210, GSE13507 cohorts were detailed in **Tables S1–3**.

### TIL Co-expression network construction

The GSVA (v1.44.1) package in R was applied to calculate the enrichment scores of every TIL set and checkpoint set in each sample of BCa utilizing a single sample gene set enrichment analysis approach (which is abbreviated as ssGSEA) (15, 16). We employed WGCNA (v1.71), a bioinformatics technique that transforms co-expression correlations into topological overlap values or connection weights, to identify co-expressed genes in TIL (17). First, the top 5,000 genes with the highest variance of TCGA-BLCA dataset and IMvigor210 dataset were chosen based on the median absolute deviation (MAD), and the gene expression files for these genes were entered into the WGCNA. Second, using the pick-Soft-Threshold function, we computed the adjacency using the soft thresholding power β, which was acquired by co-expression similarity. Third, the respective dissimilarity (1-TOM) was evaluated after the adjacency was transformed into a topological overlap matrix (TOM). Fourth, hierarchical clustering in conjunction with a dynamic tree cut function was employed to identify modules. With the purpose of classifying genes that had similar expression profiles into gene modules, average linkage hierarchical clustering was used as per the TOM-based dissimilarity measure with the lowest size (gene group) comprising 50 for the genes dendrogram. Afterward, for the modules most linked to the TIL features, module membership, as well as gene significance, were computed. Ultimately, the visualization of the eigengene network was performed. Following that, we created a Venn diagram based on the intersection of genes of the modules that were most linked to TIL in TCGA-BLCA dataset as well as IMvigor210 cohort dataset.

### Functional and pathway enrichment analysis

The intersection of the genes was investigated with the aid of gene ontology (GO) and the Kyoto Encyclopedia of Genes and Genomes (KEGG). The molecular function (MF), biological process (BP), and cellular component (CC) are the three parts of the GO enrichment analysis. KEGG provides significantly enriched mRNA-related biological pathways. The aforementioned functional enrichment analysis was conducted utilizing the clusterProfiler (v4.4.2) R package (18), the threshold was set at adjusted *P* < 0.05, and the findings were displayed using the ggplot2 (v3.3.6) R package.

### Building and verifying a risk score for anticipating the prognosis of BCa

Univariate Cox regression analysis was vital in this research in identifying the genes linked to the OS in the TCGA-BLCA cohort, and the *P*-value was established as < 0.05. The overfitting between the prognosis-linked genes was eliminated by the LASSO algorithm to minimize the extent of the prognosis-linked genes with penalty parameter tuning conducted through 10-fold cross-validation utilizing the R package “glmnet (v4.1-4)”. The genes that had non-zero regression coefficients acquired from LASSO regression analysis were included in the multivariate Cox regression analysis. The risk score was determined by the expression level of every gene multiplied by its respective regression coefficients obtained from a multivariate Cox regression analysis of individual genes. Into two risk groups, the patients were stratified in accordance with the median risk score. The Kaplan-Meier survival curves as well as the time-dependent receiver operational feature curves (ROC) that are produced by the R packages “survminer (v0.4.9)” and “survivalROC (v1.0.3)” were utilized to ascertain the risk score’s performance in anticipating BCa patients’ survival. Following that, the verification of the accuracy of the risk score model in the GSE13507 dataset was performed utilizing a similar method. Immunohistochemistry data from the Human Protein Atlas (HPA) database (https://www.proteinatlas.org) confirmed the expression of these model genes in BCa (19).

### Development and verification of the clinical prognostic model

To ascertain if the risk score is capable of functioning as an independent risk factor influencing the survival of BCa patients, univariate as well as multivariate analyses were performed on both the TCGA-BLCA dataset and GSE13507 dataset. A nomogram incorporating age, gender, stage, grade, and risk signature for survival anticipation was constructed *via* the “rms (v6.3-0)” package. On the other hand, the predictive accuracy of the nomogram was evaluated by drawing the calibration curve.

### Immune infiltration landscape analysis

The proportion of each immune cell was computed utilizing CIBERSORT and ssGSEA in order to approximate the immune infiltration landscape for two risk groups (20). To derive the proportion of 22 immune cell types that met a threshold of *P* < 0.05, the CIBERSORT (http://cibersort.stanford.edu/) algorithm was employed. Besides, the ssGSEA algorithm was used to estimate the infiltration level of 28 types of immune cells through the “GSVA” R packages (15). Additionally, the ESTIMATE, immune, and stromal scores and tumor purity were computed *via* the “ESTIMATE (v1.0.13)” R packages for the BCa patients (21).

### Calculation of tumor mutation burden and gene set enrichment analysis

The Perl scripts computed the tumor mutational burden (TMB) values from the number of variants out of the overall length of the 38 million human exons in every sample. The waterfall plots we employed in the study were derived by the “maftools (2.12.0)” R package to ascertain the number of somatic point mutations in every BCa sample (22). Furthermore, we displayed the link between risk groups and TMBs. The CNV frequencies obtained demonstrated the aforementioned findings in a lollipop chart. On the other hand, the “RCircos (v1.2.2)” package of R software was used in visualizing the gene sites on the chromosomes. To explore the potential activated pathways in the risk subgroups, GSEA was adopted (23). The annotated gene set c2.cp.kegg.v7.5.1.symbols.gmt downloading from Molecular Signatures Database (http://www.gsea-msigdb.org/gsea/index.jsp) served as the study’s reference gene sets.

### Immunotherapy response prediction

The BLCA project of The Cancer Immunome Atlas (TCIA, https://tcia.at/) was instrumental in acquiring the immunophenoscore (IPS) of BCa samples, which can predict the response to immunotherapies including CTLA4 and PD-1 blockers (24). The immunophenoscore score was normalized to 0-10. A greater IPS score signified a greater immunological reactivity. Additionally, we employed the IMvigor210 dataset to verify the connections between immunotherapy and the risk signature.

### Validation of hub genes by quantitative real-time PCR

We used qRT-PCR to verify the expression level of hub genes in BCa cell lines. Human BCa cell lines (J82 and T24) and SV-HUC-1 cells were purchased from the Cell Bank of the Chinese Academy of Sciences (Shanghai, China). All cells were cultivated in RPMI-1640 (Gibco, USA) with 10% fetal bovine serum (Gibco, USA) at 37°C in a humidified chamber with 5% CO2. Total cellular RNA was isolated using TRIzol reagent (Beyotime, China) and transcribed into cDNA using PrimeScript RT Reagent Kit (Servicebio, China) according to the manufacturer’s instructions. The expression of genes was evaluated by qRT-PCR using SYBR Green qPCR Master Mix kit (Servicebio, China) and compared using the 2^-ΔΔ^Ct method (GADPH as the internal control). The primers are summarized in **Supplementary Table S4**.

### Statistical analysis

R software (version 4.2.0, https://www.r-project.org/) conducted every statistical analysis in our study. The independent Student’s t-test, as well as the Wilcoxon rank-sum test, were adopted to compare continuous variables between two groups and between non-normally distributed variables, correspondingly. A chi-squared test was utilized to compare the categoric variable data for the two risk groups. Unless otherwise mentioned in the paper, the *P*-value ≤ 0.05 was considered statistically significant.

## Results

### Discovery of hub modules linked to TIL in BCa and intersection function analysis

**Figure 1:**
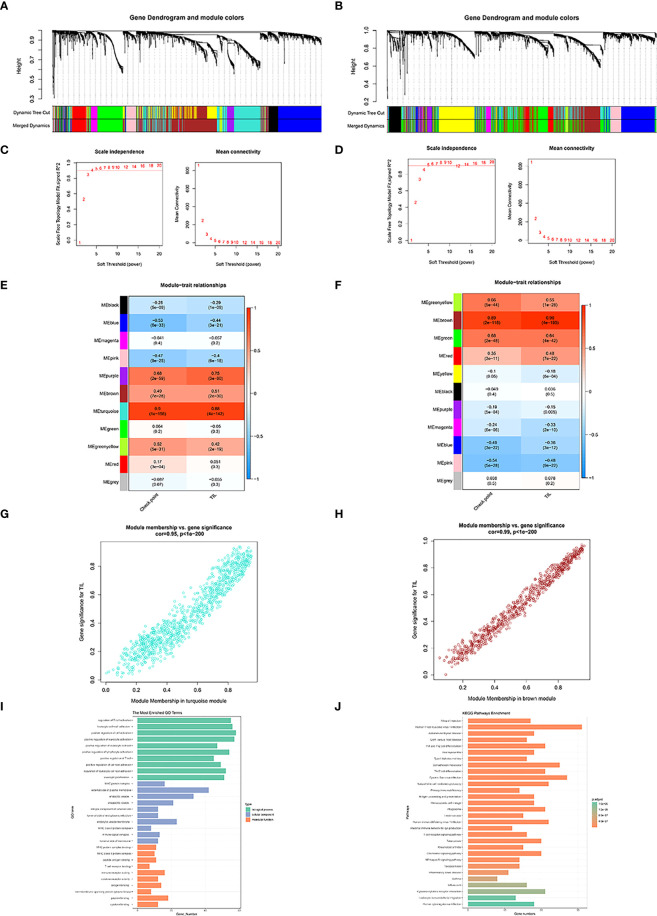
Co-expression network generated using WGCNA. Dendrogram of robust TIL clustered based on a dissimilarity measure (1-TOM) of TCGA-BLCA **(A)** and IMvigor210 dataset **(B)**. Analysis of network topology for various soft-thresholding powers of TCGA-BLCA **(C)** and IMvigor210 dataset **(D)**.Heatmap demonstrating the correlation between module eigengenes and TIL in the TCGA-BLCA **(E)** and IMvigor210 dataset **(F)**. The correlations between TCGA turquoise module membership and TIL-related genes (TILRGs) significance **(G)**. The correlation between IMvigor210 brown module membership and TILS **(H)**. Gene ontology (GO) **(I)** and Kyoto Encyclopedia of Genes and Genomes (KEGG) **(J)** analyses of TIL-related intersecting genes.

The whole flowchart of the study is shown in **Figure S1**. **Figures 1A**, **B** show the dendrogram of robust TIL clustered using a dissimilarity measure. The analysis of the scale-free fit index, as well as mean connectivity for diverse soft thresholding powers, was shown in **Figures 1C, D**, respectively. Among the 11 modules in the TCGA-BLCA dataset, the turquoise module was substantially linked to TIL (R^2^ = 0.88, *P* = 4e-142) (**Figure 1E**). The brown module was substantially linked to TIL (R^2^ = 0.96, *P* = 4e-195) (**Figure 1F**) in the IMvigor210 cohort, out of the 11 modules. Utilizing these results, we supplemented the heatmap of the link between the factors in the turquoise module and the brown module (**Figures 1G, H**).

1018 TIL co-expressed genes were identified in the TCGA-BLCA turquoise module. We furthermore detected 742 TIL co-expressed genes in the IMvigor210 brown module. Following that, 259 co-expression genes were identified with the aid of the intersection part of these two modules (**Figure S2**). GO analysis exhibited that the intersection of genes was primarily enriched in functions like the regulation of T cell activation, external side of plasma membrane, and immune receptor activity (**Figure 1I**). KEGG analysis of the intersection of genes was associated with Th1 and Th2 cell differentiation, Cell adhesion molecules, Th17 cell differentiation, Natural killer cell mediated cytotoxicity, and antigen processing and presentation (**Figure 1J**).

### Construction and validation of the TIL models

**Figure 2:**
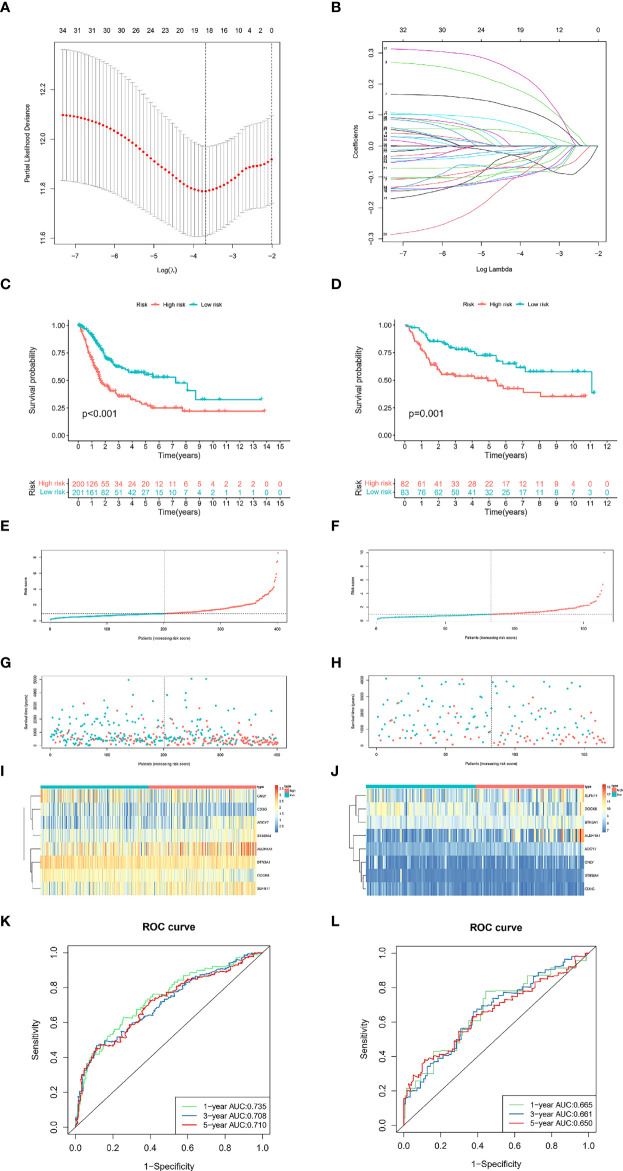
Development and validation of the TILS. The minimum standard **(A)** and the corresponding coefficient **(B)** of LASSO Cox regression analysis. Kaplan-Meier survival curves of high- and low-risk groups in the TCGA-BLCA **(C)** and GSE13507 dataset **(D)**. Red represents high risk, and cyan represents low risk. The distribution of risk score and survival status of BCa patients with increasing risk score in the TCGA-BLCA dataset **(E, G)** and GSE13507 dataset **(F, H)**. Heatmap for the expression of eight crucial genes in low- and high-risk groups in the TCGA-BLCA **(I)** and GSE13507 dataset **(J)**. ROC curves analysis of TILS on OS at 1-year, 3-years, and 5-years in the TCGA-BLCA **(K)** and GSE13507 dataset **(L)**.

To effectively implement TILRGs in the therapeutic management of BCa and compute the particular risk score of every BCa patient, we created a specific risk scoring model using TILRGs. Firstly, out of 259 TILRGs, 34 prognosis-related genes were identified by the univariate Cox regression analysis of OS (*P* < 0.05) (**Table S5**). To avoid the risk of over-fitting, the LASSO regression analysis was employed in reducing the number of candidate genes using the lowest value of lambda λ (**Figures 2A, B**). The relevant coefficient of the optimal eight TILRGs was determined *via* multivariate Cox regression analysis. Afterward, we developed a novel signature for all BCa individuals utilizing the formula risk score = 0.2619 × expression of ADCY7 + (-0.1884) × expression of DOCK8 + 0.19518 × expression of SLFN11 + 0.3188 × expression of ST8SIA4 + 0.0638 × expression of ALDH1A1+(-0.2430) × expression of CD3G+(-0.1260)×expression of GNLY + (-0.3092) × expression of BTN3A1, named the TIL-related risk score signature (TILS). The patients with BCa were grouped into low as well as high-risk groups as per the median value of risk score. Additionally, Kaplan-Meier analysis ascertained that patients belonging to the high-risk group had substantially poorer OS in contrast with those belonging to the low-risk group, as demonstrated in **Figures 2C, D**. In addition, there are significant differences in disease-free survival, disease-specific survival, and progression-free survival of TCGA cohort in high- and low-risk groups of TCGA cohort (**Figures S3A–C**). Risk curve analysis, on the other hand, manifested that the high-risk group had higher mortality and shortened survival duration (on the right side of the dotted line). **Figures 2E–H** show that as the risk score rose, the proportion of patients and deaths in the high-risk group were elevated. The heatmaps of eight crucial genes expression of TCGA-BLCA dataset and GSE13507 dataset were shown in **Figures 2I**, **J**, respectively. The AUCs for 1-, 3-, and 5-year OS were 0.753, 0.703, and 0.710, correspondingly, in the TCGA cohort (**Figure 2K**), whereas in the GSE13507 cohort they were 0.665, 0.661, and 0.650, respectively (**Figure 2L**). These findings were in total agreement with those of the TCGA cohort, implying a strong separation capability.

### Construction and validation of the nomogram and independent prognostic factor analysis

**Figure 3:**
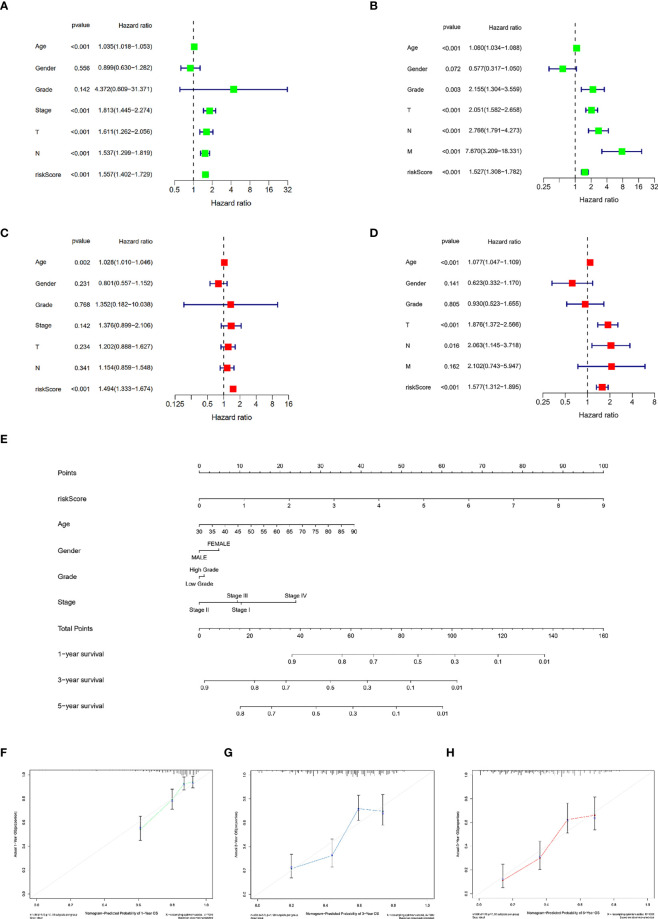
Establishment and assessment of the nomogram for survival prediction. Univariate and multivariate Cox regression analyses showed that risk score based on TILRGs is an independent prognostic factor affecting the prognosis of BCa patients in the TCGA-BLCA dataset **(A, C)**. Univariate and multivariate Cox regression analyses showed that risk score based on TILRGs is an independent prognostic factor affecting the prognosis of BCa patients in the GSE13507 dataset **(B, D)**. The nomogram combining risk score based on TILRGs and other clinicopathological parameters was developed to predict 1-, 3-, and 5-year survival **(E)**. Calibration curves showing the predictions of the nomogram that we established for 1- **(F)**, 3- **(G)**, and 5-year **(H)** overall survival.

To ascertain the clinical independence of the risk model, the central method we used was the univariate Cox regression analysis, which affirmed that the Risk Score was strongly linked with survival (**Figures 3A, B**). In addition, the Risk Score remained strongly connected with survival after correcting for biological factors, according to multivariate Cox regression analysis (**Figures 3C, D**). As evidenced by the aforementioned data, the risk model exhibits high predictive performance for clinical applications. Subsequently, we created a clinically adaptive nomogram using the TILS as well as other clinicopathological features to give a vivid presentation to anticipate 1-, 3-, and 5-year survival with BCa. Both short- as well as long-term survival was better predicted using our nomogram (**Figure 3E**). The nomogram’s calibration plot revealed outstanding consistency between the nomogram’s forecast and the actual observation probabilities (**Figures 3F–H**).

### Relationship between clinicopathological characteristics and the TILS

**Figure 4:**
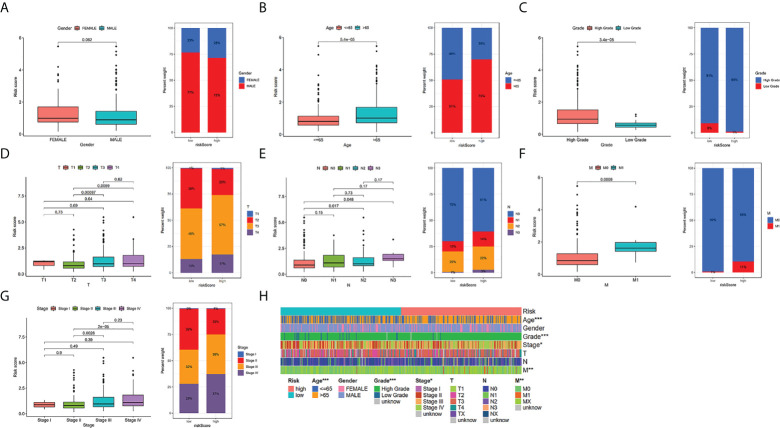
Relationship between risk scores and clinical characteristics in TCGA-BLCA cohort. Relationship between risk score and gender **(A)**, age **(B)**, grade **(C)**, T stage **(D)**, N stage **(E)**, M stage **(F)**,tumor stage **(G)**. The heatmap shows the relationship among gender, age, grade, T stage, N stage, M stage, tumor stage and the risk score **(H)**. * *P* < 0.05, ** *P* < 0.01, and *** *P* < 0.001.

**Figure 5:**
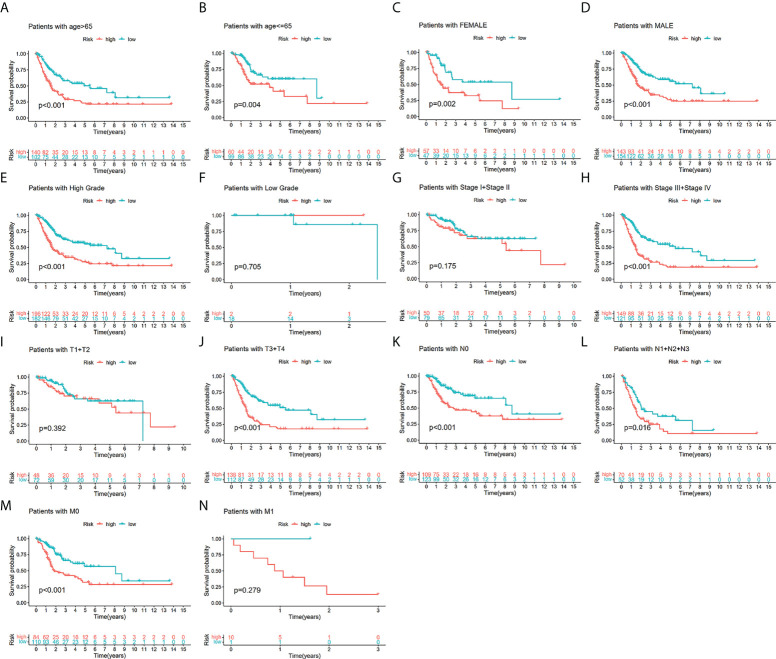
The survival outcomes of bladder cancer patients with different risk scores in subgroups of clinicopathological parameters in the TCGA-BLCA. Patients with higher risk scores had worse overall survival compared with those with lower risk scores in most subgroups forage > 65 **(A)**, age ≤ 65 **(B)**, female **(C)**, male **(D)**, low grade **(E)**, high grade **(F)**, low-stage **(G)**, advanced-stage **(H)**, low T-stage **(I)**, high T-stage **(J)**, nodal metastasis-free **(K)**, nodal metastasis **(L)**, metastasis-free **(M)** and metastasis **(N)**.

To additionally investigate if TILS was strongly linked to various clinicopathological characteristics, we discovered that the clinical features encompassing age, tumor grade and stage, T stage, N stage, and M stage were substantially linked to TILS (**Figures 4B–G**). However, the distribution difference of the risk score in gender did not show a statistical significance (**Figure 4A**). The heatmap demonstrated the distributed patterns between TILS and clinicopathological characteristics (**Figure 4H**). The age, tumor grade, M stage, and tumor stage were diversely distributed in the two risk groups. The patients with high-grade and advanced-stage of cancer had a greater probability of being in the high-risk group. Moreover, the low-risk group was substantially linked to low-grade as well as early-stage cancer, an indication of a better prognosis. We furthermore classified BCa patients into distinct groups taking into account the age, gender, tumor grade, tumor stage, and the T, N, and M stages. In most stratification categories, there were remarkable variations between the two risk groups, implying that the low-risk group had longer OS (**Figures 5A–N**).

### Tumor mutation burden evaluation in a variety of risk groups

**Figure 6:**
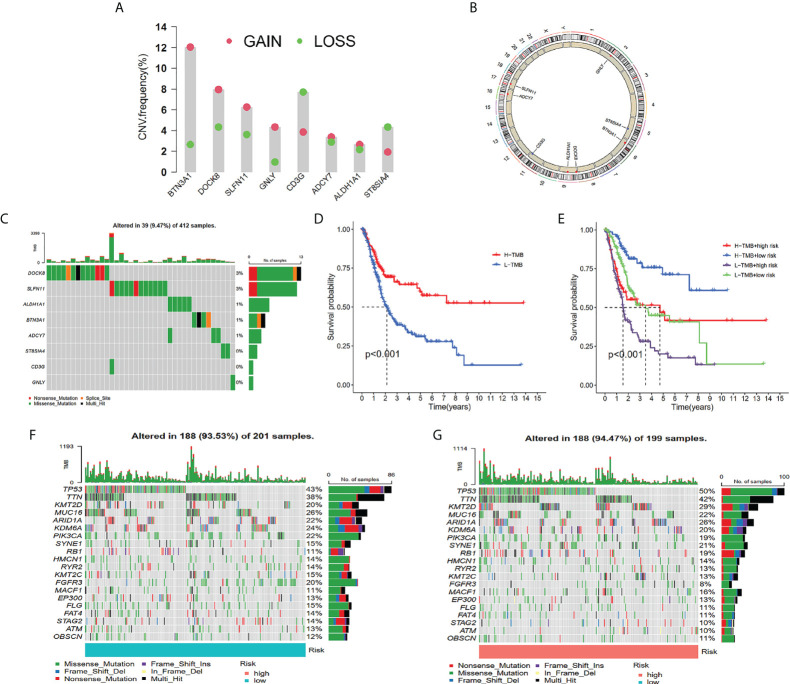
Tumor mutation analysis between different TILS groups. Frequencies of gain and loss for selected genes from the TILS **(A)**. Circus plots of chromosome distributions of selected genes from the TILS **(B)**. Gene network of TILS **(C)**. Mutation frequency of the TILS of BCa patients in the TCGA-BLCA cohort **(D)**. Kaplan-Meier curves showed that high tumor mutation burden (H-TMB) subgroup had better survival probability than Low-TMB (L-TMB) subgroup in the TCGA-BLCA **(E)**. The Kaplan-Meier curve survival analysis for BCa patients stratified by both TMB groups and TILS **(F)**. Waterfall plot displaying gene mutations in the low- **(G)** and high-risk **(H)** groups.

The frequency of genetic amplification and deletion of chosen genes from the TILS was investigated. The aforementioned findings affirmed that BTN3A1, DOCK8, and SLFN11 had an elevated frequency of gain-of-function mutations in BCa, while CD3G and ST8SIA4 had an elevated frequency of loss-of-function mutations (**Figure 6A**). **Figure 6B** demonstrates the TILS copy number circle diagram, which exhibits the location of the CNV mutation of the genes of TILS on the chromosome. The overall average mutation frequency of the genes of TILS was low, with only 39 of 412 samples having the genes of TILS mutations (**Figure 6C**).

In tumorigenesis and progression, TMB played a critical role. As a result, we characterized TMB’s important function in the risk score further. Utilizing the median TMB value, we classified the patients with BCa in the TCGA dataset into high- as well as low-TMB subgroups. As per the Kaplan-Meier survival analysis, it was ascertained that the high-TMB subgroup had a higher chance of surviving in contrast with the low-TMB subgroup (**Figure 6D**). In addition, we discovered that the risk score may better anticipate the survival probability of BCa patients in high- as well as low-TMB categories (**Figure 6E**). In TCGA-BLCA cohorts, waterfall plots showed the mutation variations in the leading 20 genes between risk groups (**Figures 6F, G**). It was ascertained that in the high-risk group, patients exhibited a remarkable mutation frequency, which was in agreement with the TMB score. TP53 was the most prevalent mutation gene, followed by TTN, and the missense mutation was the most prevalent mutation type (**Figures 6F, G**).

### Link between risk scores and immune infiltration landscape

**Figure 7:**
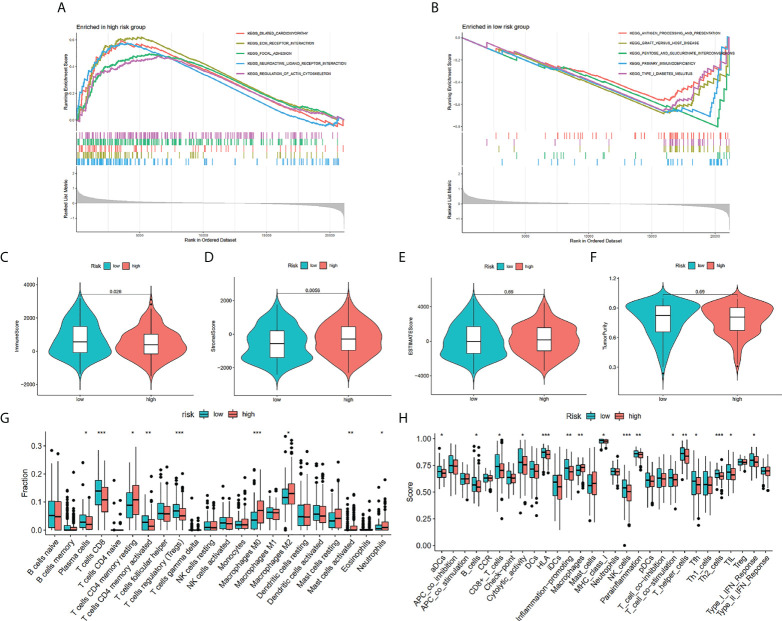
Enrichment analysis and immune estimation in high- and low-risk groups. Enrichment plots from gene set enrichment analysis in the high-risk group **(A)** and low-risk group **(B)** according to risk score based on TILRGs. Immune score **(C)**. Stromal score **(D)**. ESTIMATE score **(E)**. Tumor purity **(F)**. The fraction of 22 immune cells in low- and high-risk groups calculated by CIBERSORT algorithm **(G)**. The fraction of immune cells and immune function in low- and high-risk groups calculated by ssGSEA algorithm **(H)**. * *P* < 0.05, ** *P* < 0.01, and *** *P* < 0.001.

KEGG functional enrichment was conducted utilizing the GSEA, which discovered that the high-risk group was enriched in the process of the extracellular matrix (ECM)-receptor interactions, focal adhesion, and neuroactive ligand-receptor interaction (**Figure 7A**), and the low-risk group were enriched in the process of the antigen processing and presentation, pentose and glucuronate interconversions, and primary immunodeficiency (**Figure 7B**). We investigated the TME in the two risk groups of patients with BCa by adopting several immune assessment algorithms. The ESTIMATE findings affirmed that low-risk patients with BCa had reduced immune scores and high-risk patients had increased stromal scores. However, the ESTIMATE score and the tumor purity were not significantly different across risk groups (**Figures 7C–F**). Afterward, the CIBERSORT algorithm evaluated the immune infiltration in patients from both risk groups (**Figure 7G**). The findings affirmed that the fractions of T cells CD8, T cells CD4 memory activated, Plasma cells, and T cells regulatory (Tregs) were elevated in the low-risk group in contrast with that in the high-risk group. Nevertheless, the fractions of T cells CD4 memory resting, Mast cells activated, Macrophages M0, Macrophages M2, and Neutrophils were reduced in the low-risk group than the low-risk group. The immune infiltration utilizing the ssGSEA algorithm affirmed that the low-risk group had a remarkably greater fraction of immune cells in contrast with the high-risk group (**Figure 7G**). We identified that the proportions of B cells, CD8+ T cells, NK cells, T helper cells, T cell co-stimulation, type 2 T helper cells, T follicular helper cells, and TIL were remarkably elevated in the low-risk group in contrast with the high-risk group. Nevertheless, the fractions of Macrophages were lowered in the low-risk group in contrast with the other risk group (**Figure 7H**).

### The role of risk score in anticipating immunotherapeutic response and benefits

**Figure 8:**
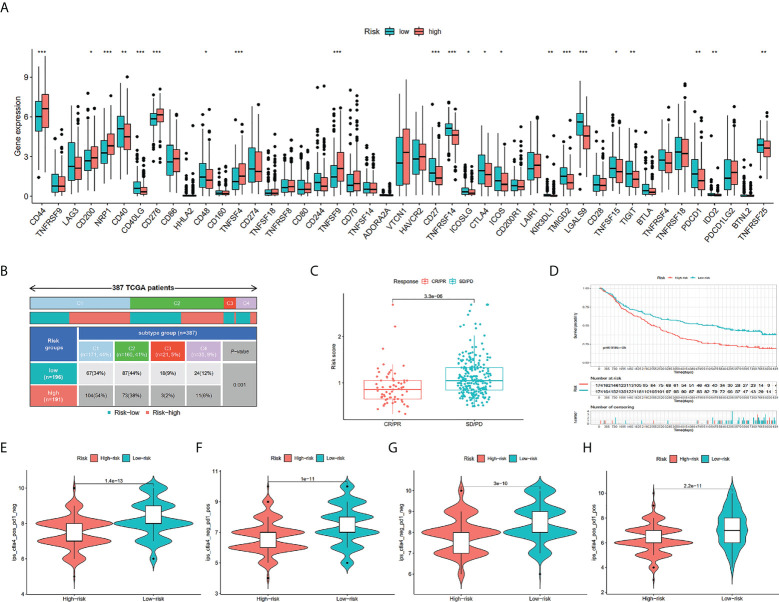
Comparison of the risk groups in our study with immune checkpoint genes and existing immune subtype and prediction of response to immunotherapeutic agents for different risk groups. The boxplot displaying the difference in immune checkpoint genes between different risk groups **(A)**. Comparison of the differences in immune subtype between different risk groups **(B)**. Comparison of risk scores between the CR/PR group and the SD/PD group **(C)**. The Kaplan-Meier curve survival analysis between the high- and low-risk groups in the IMvigor210 cohort **(D)**. CTLA4_pos_PD1_neg **(E)**, CTLA4_neg_PD1_pos **(F)**, CTLA4_neg_PD1_neg **(G)**, and CTLA4_pos_PD1_pos **(H)**. The comparison of immunophenoscore (IPS) between different risk groups. * *P* < 0.05, ** *P* < 0.01, and *** *P* < 0.001.

We assessed risk score differences in multiple checkpoint molecules. It’s worth noting that we discovered statistically significant changes in the important immune checkpoint molecules TIGIT, CTLA4, and PDCD1 between individuals in the two risk groups, deducing that the immunotherapy response rate in them may differ (**Figure 8A**). As depicted in **Figure 8B**, there were more C3 and C4 subtypes in the low-risk score subgroup; in contrast, there were more C1 subtypes in the high-risk score subgroup (*P* = 0.001). In a urothelial cancer cohort that was treated with anti-PD-L1 antibody atezolizumab, IMvigor210, the TILS was validated. The result affirmed that risk score was lowered in immunotherapy complete/partial response patients (**Figure 8C**). Furthermore, we discovered remarkably elevated survival chances in the high-risk score group in contrast with the other subgroup (**Figure 8D**). TCIA findings also attained a similar conclusion. The finding indicated that the low-risk group had greater TCIA scores (CTLA-4_pos_PD-1_neg, CTLA-4_neg_PD-1_neg, CTLA-4_pos_PD-1_pos, and CTLA-4_neg_PD-1_pos) in contrast with the high-risk group, implying that low-risk can have an enhanced immunotherapy potency of anti-CTLA4 as well as anti-PD-1 therapy (**Figures 8E–H**).

### The results of qRT-PCR and immunohistochemistry

We explored the expression of TILRGs at the cellular level. qRT-PCR results suggested that BTN3A1 and ST8SIA4 expression levels were significantly increased in BCa cell lines (**Figures S4F, G**), while the relative expressions of SLFN11, DOCK8, ADCY7, GNLY, and CD3G expression were reduced in BCa cell lines (T24 or J82 cells) compared with the human uroepithelial SV-HUC-1 cell line (**Figures S4A–E**). Additionally, the expression of ALDH1A1 varies greatly in BCa cell lines but is not stable. The immunohistochemical results of the expression of the 8 genes mentioned above in BCa and normal tissues from the HPA database were shown in **Figures S5A–H**, which were mostly consistent with qRT-PCR results.

## Discussions

BCa is one of the most prevalent urinary tract carcinomas all over the globe. There are about 550,000 new cases every year, and the number of cases is increasing year by year (1). The 5-year survival rate for patients in the USA is 77% and 5% for patients with metastatic bladder cancer (25). Due to its characteristics of easy recurrence and progression, comprehensive and individualized treatment of BCa is still a challenge for clinicians. Recently, with our in-depth knowledge of the immunopathological mechanism and TME of BCa, immunotherapy is becoming a novel treatment option for patients suffering from BCa. Previous research discovered that TIL has a central function in tumor therapy, especially immunotherapy, and is associated with the responses to blockade of the immune checkpoint (26–28). It was disclosed that immunotherapy response rates in patients that had PD-L1-positive tumors and TIL ranged from 29% to 43%, which means many patients with enhanced PD-L1 expression fail to respond to treatment (29). Understanding the key factors of immune checkpoint blockade is particularly important for screening patients who respond to immunotherapy. More importantly, several clinical studies have affirmed that TIL and PD-1 are closely linked to the poor prognosis of BCa (30, 31). Consequently, it is vital to systematically explore gene mutation status, clinical features, prognosis, immune cell infiltration, immune subtype, TME, and immunotherapy response between different risk subgroups based on TILS.

In this investigation, we first applied WGCNA to discover a hub module gene associated with immune checkpoints and tumor-infiltrating lymphocytes in BCa from TCGA-BLCA dataset and IMvigor210 dataset. Through GO as well as KEGG analyses, it was discovered that these co-expression genes of the hub module were enriched in some typical immune-associated signaling pathways. These signaling pathways engage various core biological processes, the majority of which are linked to the modulation of immunity, and a few have been determined to be linked to immunotherapy. This suggests the significance of the intersection of genes and avails the basis for conducting a relationship analysis between the intersection of genes and immunophenotypes. This suggests that these genes might be engaged in the underlying mechanisms of BCa progression and immune evasion. Subsequently, univariate Cox, LASSO Cox regression, and multivariate Cox analysis were performed on these common genes. Finally, 8 hub genes were identified to establish prognostic risk models. We explored differences in the expression of these 8 genes between tumor and paracancer at the cellular and tissue levels. Among the eight genes, five of the eight immune-related genes (SLFN11, DOCK8, ADCY7, GNLY, and CD3G) were lowly expressed in the BCa, while two immune-related genes (BTN3A1 and ST8SIA4) were highly expressed. Prior studies affirmed that these genes were closely linked to the occurrence, progress, and TME of carcinoma. For example, Payne et al. have found that anti-CD277 antibodies transform BTN3A1 from an immunosuppressive molecule to an immunostimulatory molecule. Then, BTN3A1 could dynamically coordinate αβ and γδ T-cell-driven antitumor immunity to eliminate the malignant progression of ovarian cancer (32). Additionally, a few studies have revealed that BTN3A1 is a candidate prognostic biomarker for pancreatic adenocarcinoma, metastatic gastrointestinal stromal tumor, head and neck squamous cell carcinoma, and renal cell carcinoma (33–35), and can effectively anticipate response to nivolumab therapy among patients with metastatic renal cell carcinoma (36). It has been reported that SLFN11, a cellular restriction factor, is closely related to tumor immune lymphocytes, immune checkpoint genes, chemokines, and immune-related signaling pathways in clear cell renal cell carcinoma (37), which is similar to our bioinformatics analysis. In addition, SLFN11, as a general target, is considered to exert a vital regulatory function in improving the sensitivity of multiple malignancies to chemotherapy, such as ovarian cancer, prostate cancer, small cell lung cancer, and so on (38–40). Taniyama et al. found that positive expression of SLFN11 can predict better OS in advanced BCa patients treated with platinum-based chemotherapy. However, SLFN11 had the opposite effect on OS in BCa patients who did not receive platinum-based chemotherapy (41). Due to its capacity to control lymphocyte migration, survival, and immune synapse formation, DOCK8 is essential for immune surveillance (42, 43).

We then established and verified a novel eight immune-related genes prognostic risk model that can accurately anticipate the survival of BCa patients. Further, eight hub genes were utilized to derive the risk score of the BCa patients, which were classified into the two risk groups. The findings ascertained that the risk score of immune-related genes signature is an independent prognostic factor. Moreover, individuals having low-risk scores experienced better disease-specific survival as well as progression-free survival compared to individuals with high-risk scores in two cohorts. We also discovered that the risk scores may be significantly associated with several clinical factors, comprising grade, T stage, N stage, and M stage. It is well understood that high stage and low grade were poor prognosis factors (44, 45), so we can believe that these factors are associated with immunity, which is consistent with our analysis results. Moreover, we constructed a nomogram comprising clinicopathological parameters such as age, gender, grade, stage, and the risk score in order to anticipate the probabilities of 1-, 3- and 5-year survival. Our results of calibration charts indicated that the nomogram has a better predictive performance for the BCa patients’ actual survival prognoses, suggesting that the TILS had broad application and practicality for prognosis prediction.

CNV, including gene gain, amplification, deletion, and loss, often significantly affects the expression levels of various oncogenic or tumor suppressor genes (46). It has been reported that frequent CNV is a vital molecular process for the occurrence and development of cancer and might change the TME and immune infiltration state (47–49). Our analysis showed that the oncogenic driver gene BTN3A1 had the highest incidence of CNV (12%), mainly gene gain. Recent studies have shown that BTN3A1 is essential to the prenyl pyrophosphate-mediated activation of Vγ9/Vδ2 T-cells and plays a fundamental function in human cancer immunotherapy (50, 51). Additionally, the types of mutations in these 8 genes were mainly missense mutations, which may also affect gene expression. TMB is remarkably linked to the effectiveness of PD-1/PD-L1 inhibitors and is widely regarded as a predictive biomarker of immunotherapy response to a certain extent (52, 53). We investigated the difference in TMB between the two risk groups. Our study found that a substantial variation in the survival time existed in both high- and low-TMB patients. Moreover, TP53, as a tumor suppressor gene, is frequently mutated in various human malignancies (54). Notably, the frequency of TP53 gene mutations was elevated in high-risk groups in contrast with low-risk groups, because the gene mutation may promote tumor cells to express more neoantigens, and thus be more likely to elicit immune responses (55, 56).

Through the KEGG analysis between the high- as well as low-risk groups, we identified that immune-related pathways and immune disease were primarily enriched in the low-risk group, such as antigen processing and presentation, graft versus host disease, and so on. Furthermore, utilizing the ESTIMATE algorithm, we ascertained the differences in TME cell infiltration between the two risk groups. The findings show that these two groups had varied TME characteristics. The findings affirmed that the low-risk group of BCa was linked to greater immune scores and lower stromal scores. Additionally, we carried out a differentiation of the contents of immune cells in two risk groups utilizing the CIBERSORT and ssGSEA. The findings determined that the high-risk group had substantially elevated infiltrative levels of M0 macrophage, M2 macrophage, T cells CD4 memory resting, and mast cells activated, which is in agreement with prior studies (57, 58). Investigations have shown that in most tumor microenvironments, M2 macrophages are engaged in inflammation resolution and suppress tumor cell immunity, thereby promoting cancer progression and metastasis (59, 60). For example, heat shock factor 1 can not only promote macrophage infiltration *via* CCL20, but also strongly correlate with lymphatic metastases and a poor outcome in BCa (61). This suggests that M2 macrophages may be associated with poor prognosis in BCa.

Immune checkpoints are a class of programmed cell death receptors and ligands that modulate the intensity and degree of the immune response, maintain the immune balance of the human body, and prevent autoimmune injury (62). Immunotherapy using ICI is currently a research hotspot in the field of tumor therapy and has a central function in various malignant tumor immunotherapy. Zhang et al. demonstrated that WD repeat domain 5 (WDR5) markedly correlated with PD-L1 expression in BCa and that OICR-9429, the WDR5 inhibitor, suppressed immune evasion by blocking PD-L1 activation induced by IFN-γ (63). The expression levels comparisons of immune checkpoints with their ligands in our analysis between the two risk score groups ascertained that the expression levels of CD44, CD200, NRP1, CD276, TNFSF4, and TNFSF9 were elevated in the high-risk group, whereas the expression levels of other immune checkpoint genes were lowered in the low-risk group. Additionally, we found that immune subtype C1 (wound healing) was particularly dominant in the high-risk group (54%). And the proportions of the C1 subtype (wound healing) were remarkably raised in the high-risk group in comparison with the low-risk group, while the result of C3 was the converse. Past studies have reported that immune inflamed subtypes have the best response to ICI treatments and can gain benefit from immunotherapy (such as anti-PD-1 and anti-CTLA-4) (64, 65). It is noteworthy that patients with stable disease and progressive disease had greater immune scores than patients with complete remission and partial remission. Similarly, the Kaplan-Meier curves depicted that the elevated immune score was substantially correlated with shorter OS of the BCa patients. Furthermore, we examined the response of the high- and low-risk groups to the four immunotherapy subtypes (CTLA-4_pos_PD-1_neg, CTLA-4_neg_PD-1_neg, CTLA-4_pos_PD-1_pos, and CTLA-4_neg_PD-1_pos). The results demonstrated that the relative probabilities of ICI treatment response were remarkable in the low-risk group, which also deduced that patients with low immune scores may be candidates for ICI therapy and our TILS model can help to guide immunotherapy in BCa patients.

Nevertheless, there are some shortcomings to this study. First, we explore TILRGs and verify TILS characteristics using bioinformatics methods and public data sets. More basic research is needed on the molecular mechanism between TILRGs and tumor immunity, although we verified the expression of these eight immune-related genes in BCa cell lines. Second, this study provides a new therapeutic target for immunotherapy in BCa patients, but its potential value in the real world remains to be further investigated. Third, there are limited clinicopathological variables available in public databases, so the data involved in prognostic and immunotherapeutic analyses may not be comprehensive enough. Clinical samples will be needed to validate these results in the future.

In conclusion, this study discovered TILRGs in BCa patients using WGCNA; additionally, we constructed and verified a robust and integrated model to anticipate the prognostic state of BCa patients, and it demonstrated a better predictive ability. We comprehensively evaluated the variation in clinicopathological features, CNV, TMB, immune microenvironment, and immunotherapy response between high- and low-risk groups. The aforementioned results may contribute to enhancing our knowledge and comprehension of the features of TIL infiltration, developing novel targets for immunotherapy of BCa, and providing important evidence for personalized therapy in the future.

## Data availability statement

The original contributions presented in the study are included in the article/**Supplementary Material**. Further inquiries can be directed to the corresponding authors.

## Author contributions

ZH, JG, MD, and YH conceived and designed the study. ZH, JG, TL, HL, CL, ZC, and EL analyzed the data and organize figures and tables. All authors helped to interpret the results. ZH and JG wrote the draft of the paper. CL, JW, MD, and YH reviewed the manuscript. MD and YH gave administrative support, fund acquisition, and supervision. All authors contributed to the article and have approved the submitted manuscript.

## Funding

This study was supported by the Applied Basic Research of Yunnan Province - Kunming Medical Joint Special Project [2019FE001 (–226)], the Construction Project of Innovation Team of Colleges and Universities in Yunnan Province (2019GXCXTD01), Famous Doctor Project of “Ten Thousand People Plan” in Yunnan Province (RSC2020MY024), Leading Talents Program of Yunnan Province (L-2018009), the Yunnan Provincial Education Department Scientific Research Fund Project (2022J0220), the Yunnan Fundamental Research Projects (202201AU070201), the Project of Kunming Medical University Graduate Innovation Fund (2022S068) and the National Natural Science Foundation of China (81972390).

## Acknowledgments

This study used and analyzed currently publicly available datasets. We appreciate the generous sharing of data from the Cancer Genome Atlas (TCGA) database, the Gene Expression Omnibus (GEO) database, and the website based on the Creative Commons 3.0 license.

## Conflict of interest

The authors declare that the research was conducted in the absence of any commercial or financial relationships that could be construed as a potential conflict of interest.

## Publisher’s note

All claims expressed in this article are solely those of the authors and do not necessarily represent those of their affiliated organizations, or those of the publisher, the editors and the reviewers. Any product that may be evaluated in this article, or claim that may be made by its manufacturer, is not guaranteed or endorsed by the publisher.

## Supplementary material

Supplementary Figure 1 The whole flowchart of the study.

Supplementary Figure 2 The Venn plot of 259 intersection TILRGs.

Supplementary Figure 3 The Kaplan-Meier curve survival analysis of disease free survival **(A)**, disease-specific survival **(B)**, and progression free survival **(C)** in the high- and low-risk groups.

Supplementary Figure 4 qRT-PCR results of eight TILRGs expression levels. **(A)** GNLY, **(B)** SLFN11, **(C)** DOCK8, **(D)** CD3G, **(E)** ADCY7, **(F)** ST8SIA4, **(G)** BTN3A1, **(H)** ALDH1A1. ns not signifcance, * *P* < 0.05, ** *P* < 0.01, and *** *P* < 0.001.

Supplementary Figure 5 Immunohistochemistry showing 8 genes expression levels of BCa patients in HPA database. **(A)** ADCY7, **(B)** ALDH1A1, **(C)** BTN3A1, **(D)** CD3G, **(E)** DOCK8, **(F)** GNLY, **(G)** SLFN11, **(H)** ST8SIA4.
