# Supplementary figures and images for: Comprehensive analyses of a tumor-infiltrating lymphocytes-related gene signature regarding the prognosis and immunologic features for immunotherapy in bladder cancer on the basis of WGCNA
Source: Front Immunol. 2022 Sep 20;13:973974. doi: 10.3389/fimmu.2022.973974 (PMC9540212; doi:10.3389/fimmu.2022.973974)

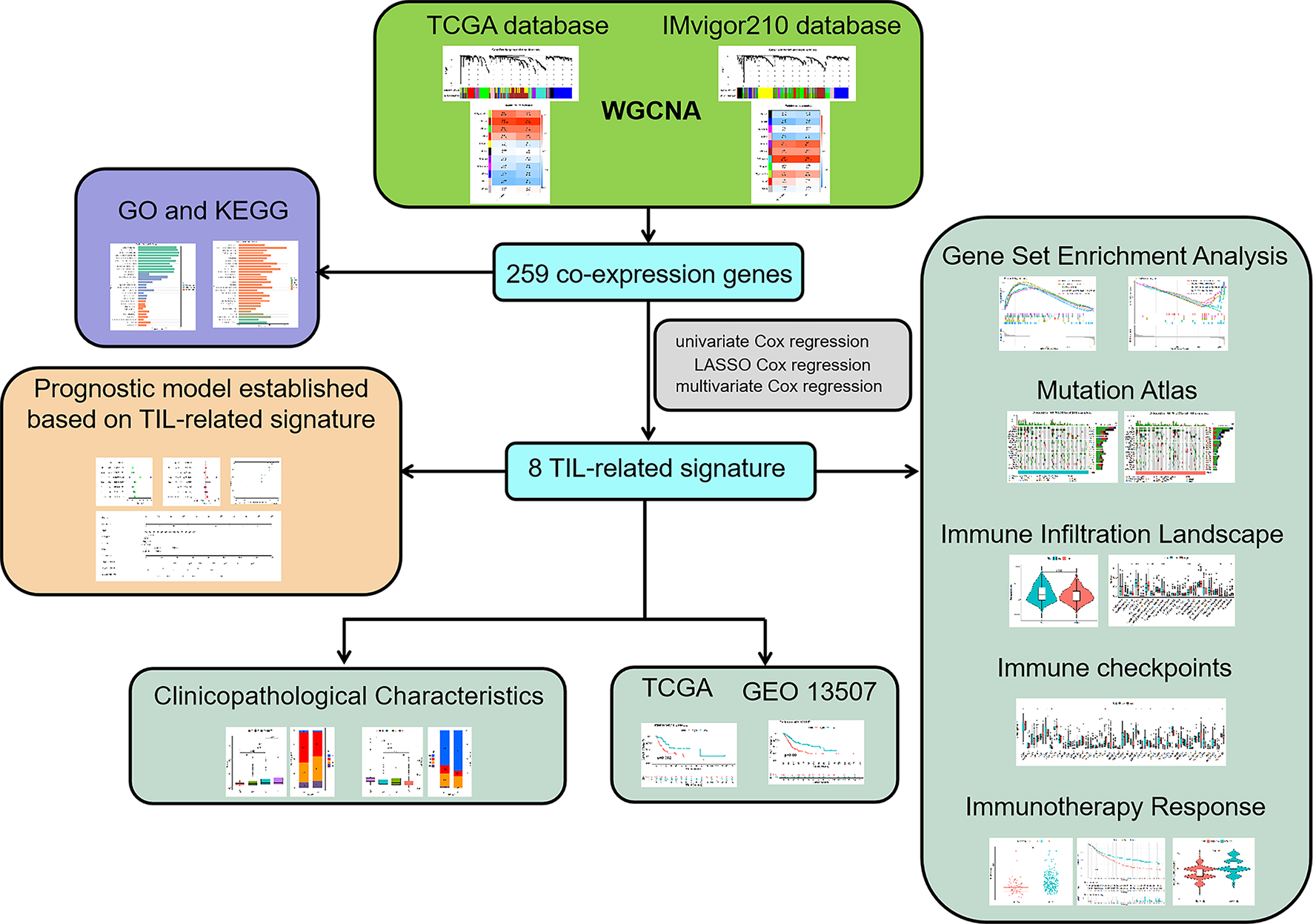

Supplement: Supplementary Figure 1 — The whole flowchart of the study. [file Image_1.tif]

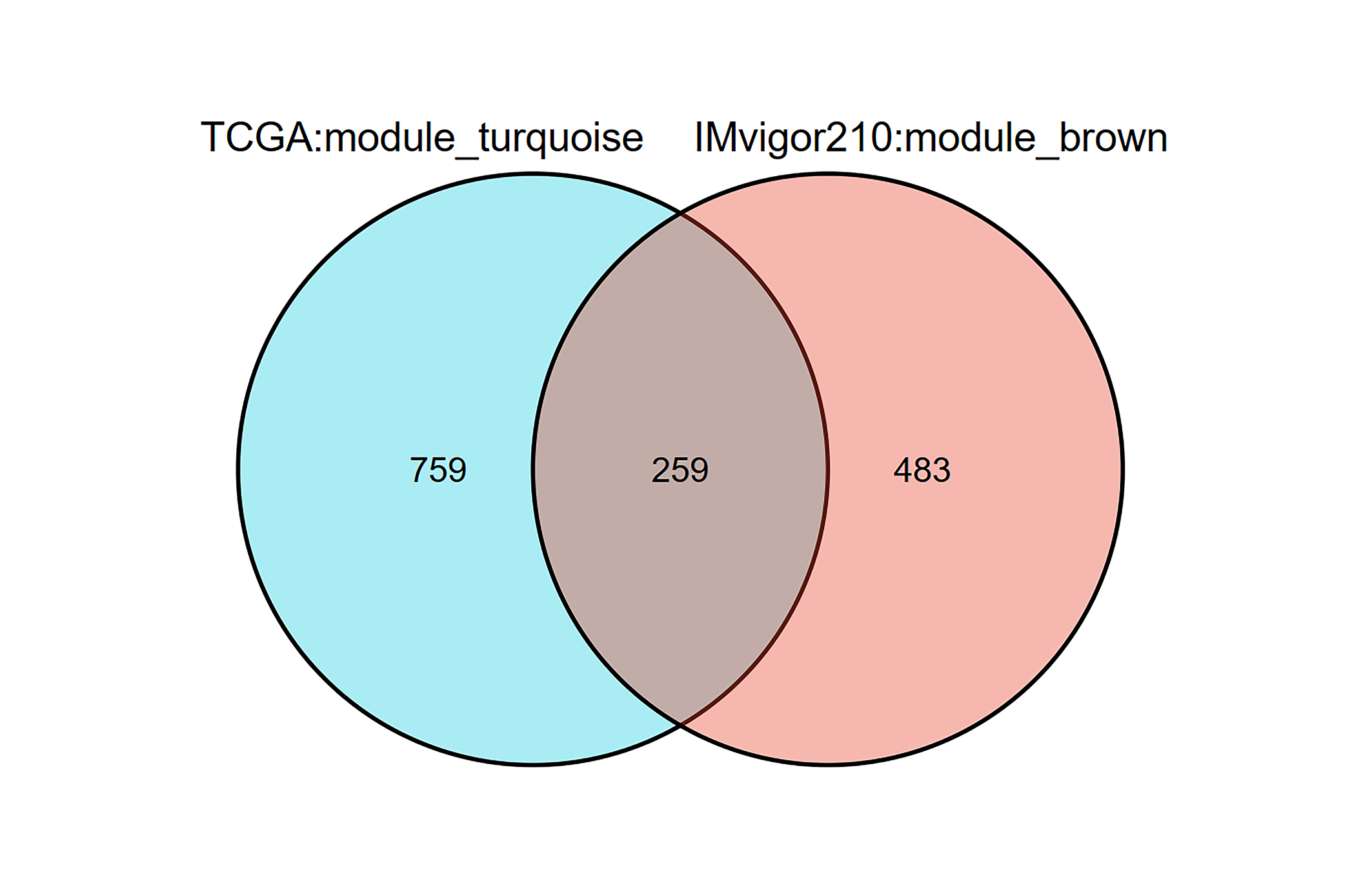

Supplement: Supplementary Figure 2 — The Venn plot of 259 intersection TILRGs. [file Image_2.tif]

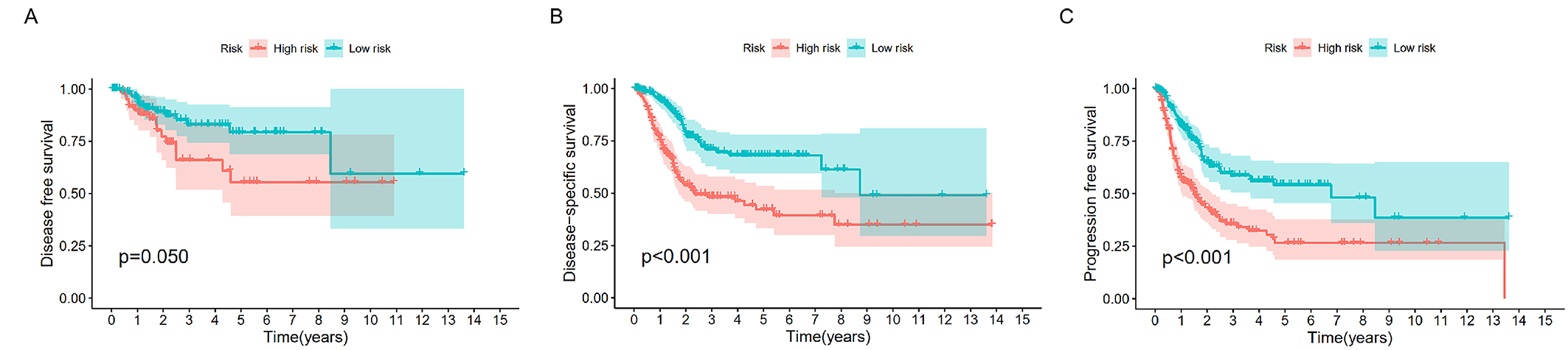

Supplement: Supplementary Figure 3 — The Kaplan-Meier curve survival analysis of disease free survival (A), disease-specific survival (B), and progression free survival (C) in the high- and low-risk groups. [file Image_3.tif]

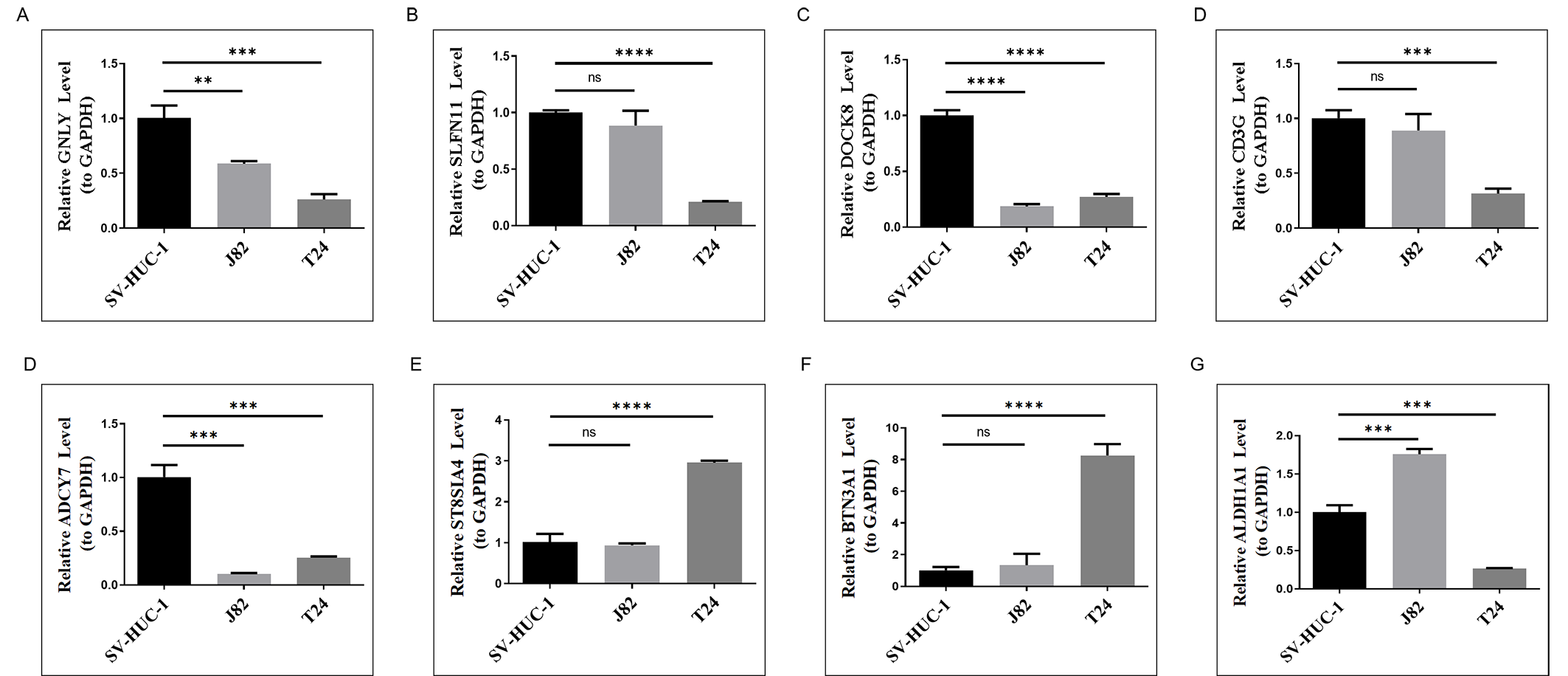

Supplement: Supplementary Figure 4 — qRT-PCR results of eight TILRGs expression levels. (A) GNLY, (B) SLFN11, (C) DOCK8, (D) CD3G, (E) ADCY7, (F) ST8SIA4, (G) BTN3A1, (H) ALDH1A1. ns not signifcance, * P < 0.05, ** P < 0.01, and *** P < 0.001. [file Image_4.tif]

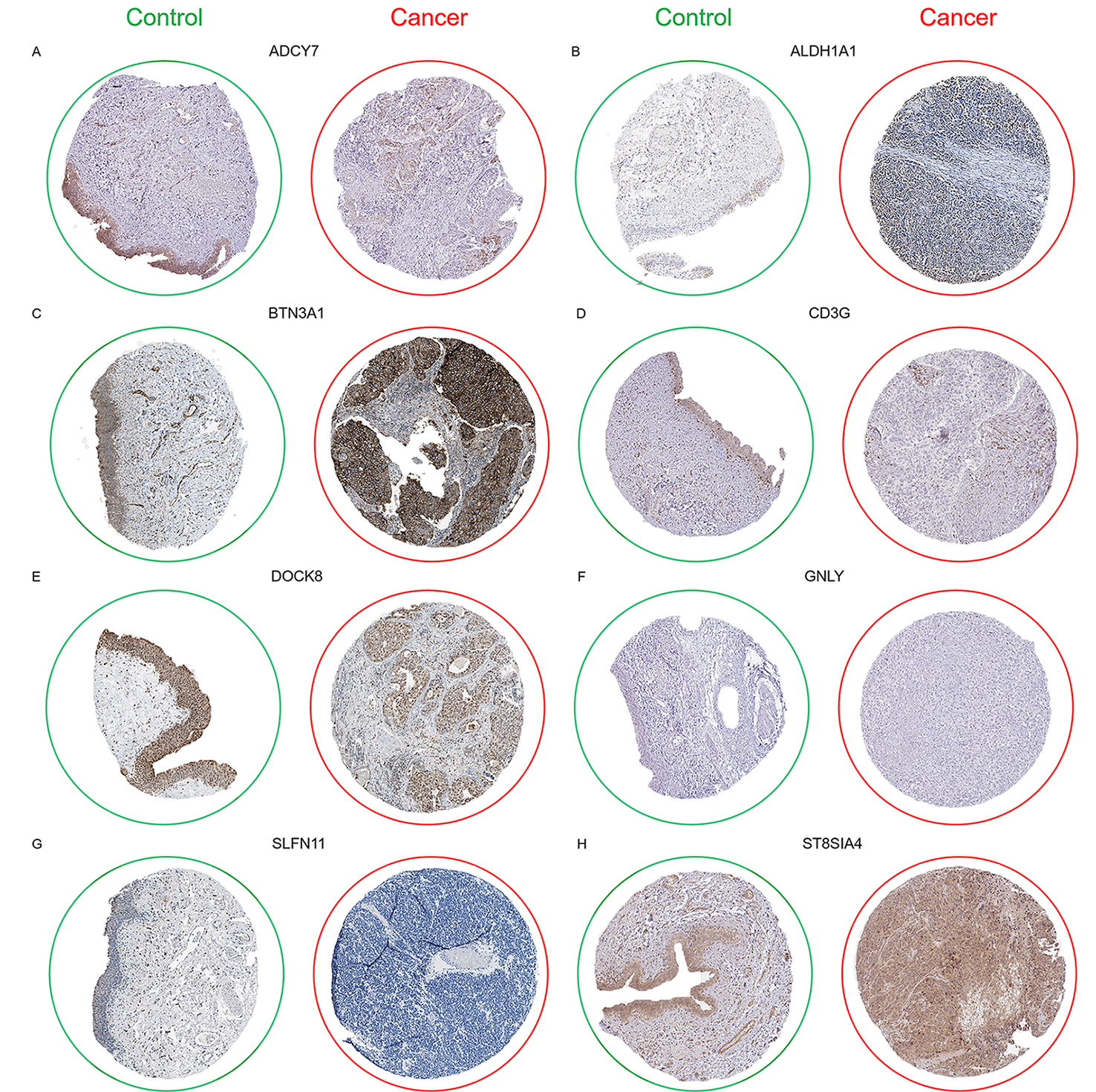

Supplement: Supplementary Figure 5 — Immunohistochemistry showing 8 genes expression levels of BCa patients in HPA database. (A) ADCY7, (B) ALDH1A1, (C) BTN3A1, (D) CD3G, (E) DOCK8, (F) GNLY, (G) SLFN11, (H) ST8SIA4. [file Image_5.tif]
